# Risk Factors Associated With Medication Errors Among Patients Suffering From Chronic Disorders

**DOI:** 10.3389/fpubh.2020.531038

**Published:** 2020-11-19

**Authors:** Muhammad Fawad Rasool, Anees ur Rehman, Imran Imran, Sameen Abbas, Shahid Shah, Ghulam Abbas, Irfanullah Khan, Sadia Shakeel, Mohamed Azmi Ahmad Hassali, Khezar Hayat

**Affiliations:** ^1^Department of Pharmacy Practice, Faculty of Pharmacy, Bahauddin Zakariya University, Multan, Pakistan; ^2^Department of Clinical Pharmacy, School of Pharmaceutical Sciences, University Sains Malaysia, Penang, Malaysia; ^3^Department of Pharmacology, Faculty of Pharmacy, Bahauddin Zakariya University, Multan, Pakistan; ^4^Department of Pharmacy, Quaid e Azam University, Islamabad, Pakistan; ^5^Department of Pharmacy Practice, Faculty of Pharmaceutical Sciences, Government College University, Faisalabad, Pakistan; ^6^Department of Pharmaceutics, Faculty of Pharmaceutical Sciences, Government College University, Faisalabad, Pakistan; ^7^Department of Pharmacy Practice, Dow University of Health Sciences, Karachi, Pakistan; ^8^Institute of Pharmaceutical Sciences, University of Veterinary and Animal Sciences, Lahore, Pakistan

**Keywords:** medication errors, drug-drug interactions, inappropriate prescribing, chronic diseases, risk factors

## Abstract

**Introduction:** Medication error is unintentional and can be reduced by reducing the risk factors. Patients suffering from chronic diseases are at an increased risk of medication errors.

**Objective:** This work aims to assess the risk factors associated with medication errors among patients suffering from chronic disorders in hospitals of South Punjab, Pakistan.

**Methodology:** Multiple logistic regression analysis was used to assess the impact of different risk factors on the prevalence of medication errors in patients suffering from chronic diseases.

**Results:** A greater risk for the occurrence of medication errors was associated with age ≥60 years (odds ratio, OR = 1.9; 95% CI = 1.3–3.1; *p* = 0.001), overburdened healthcare system (OR = 2.2; 95% CI = 1.64–3.56; *p* < 0.000), number of prescribed drugs ≥5 (OR = 1.74; 95% CI = 1.02–2.64; *p* < 0.000), comorbidities (OR = 2.6; 95% CI = 1.72–3.6; *p* = 0.003), Charlson comorbidity index (OR = 1.31; 95% CI = 0.49–1.84; *p* = 0.004), and multiple prescribers to one patient (OR = 1.12; 95% CI = 0.64–1.76; *p* = 0.001).

**Conclusion:** Older age, overburdened healthcare system, number of prescribed drugs, comorbidities, Charlson comorbidity index, and multiple prescribers to one patient are significant risk factors for the occurrence of medication errors.

## Introduction

A medication error may be defined as “failure in drug therapy that may result in harmful effects to patients.” It is a serious concern especially in low- to middle-income countries and can result in an increased risk of drug–drug interactions, elevate the frequency of hospital admissions and outpatient visits, prolong the length of hospital stay, increase the management cost, and elevate the mortality risk (1–3). Medication errors affect seven million patients annually and result in ~3.5 million doctor office visits and one million emergency department visits each year. According to the Florida Health Care Coalition, around 500,000 medication errors have been estimated daily in America. It is the third leading cause of outpatient visits after cardiovascular diseases and cancer (4). In the United States of America, more people die due to medication errors than traffic accidents, auto-immunodeficiency syndrome, or breast cancer (5, 6). Medication-related errors are responsible for ~5 to 41.3% of all hospital admissions and 22% readmissions after discharge worldwide (7).

Medication errors result in substantial economic burden on patients and the healthcare system due to an increase in the cost of treatment (8). The economic burden due to inappropriate prescribing is preventable, and controlling medication errors can result in substantial relief to the healthcare system (9). In the USA, the economic burden due to medication errors was reduced from US$ 177.4 billion in 2001 to US$ 21 billion in 2014 due to the implementation of effective strategies to control medication errors (3, 6). In the UK, before the establishment of a “system for reporting and preventing errors” and the National Patient Safety Agency (NPSA), ~850,000 adverse events were reported every year, which result in a huge economic burden of £2 billion per year due to additional hospital admissions (10). The establishment of a “reporting system” and the NPSA resulted in 40% reduction in medication errors by collecting data on adverse events and finding and implementing solutions to reduce them (11).

Different studies reported 5.4 to 66% medication errors in prescribed therapy in outpatients and hospital-admitted patients (12–14). The difference in percentage of the medication errors in different studies is due to the characteristics of patients, study environment, study settings, methods utilized for assessing and classifying the errors, and prescribing practice. The risk of medication errors (having an unnecessary drug, a non-optimal drug, or a non-optimal dose) increases with the increase in the number of drugs, comorbidities, multiple prescribers, and older age (8, 15). The risk of incidence of medication errors increased up to 30% in patients who received five or more drugs and 38% in patients with age 75 years or older (16). The medication-related admissions were almost double in older patients (65 years or above) as compared to the younger ones (17).

Chronic diseases cause substantial economic burden and are major causes of morbidity and mortality (18–20). Two-third of the chronic disease patients are older than 60 years, suffer from different comorbidities, and are on polypharmacy (five or more drugs) (21). Older age, multiple comorbidities, and polypharmacy increases the risk of medication errors in chronic disease patients (21, 22). Moreover, in low- to middle-income countries like Pakistan, inappropriate prescribing, overburdened healthcare system, lack of disease surveillance system, shortage of healthcare staff, and absence of a clinical pharmacist in most of the settings are additional risk factors for medication errors (23).

Finding the factors associated with medication errors and effective strategies to prevent errors can help healthcare professionals to identify and manage medication errors. It can also reduce the economic and humanistic burden due to medication errors. Few studies assessed the relationship between medication errors and specific factors such as patient age, number of prescribers, frequency of hospitalization, length of hospital stay, cost of hospitalization, mortality rate, number of drugs prescribed, and review of prescription by a pharmacist or specialist (24–27). Moreover, data on medication errors are mostly available from hospitalized patients (15, 28). To the best of our knowledge, no study assessed the incidence and causes of medication errors in chronic disease patients visiting outpatient clinics for regular follow-up. Thus, the objective of the present study was to assess the prevalence and the risk factors associated with medication errors among patients suffering from chronic disorders in hospitals of South Punjab, Pakistan.

## Methodology

### Study Design

This study was a cross-sectional, observational, prospective one that included 803 patients from outpatient medical wards of 17 public and private hospitals (teaching and general hospitals) in different areas of South Punjab, Pakistan. The hospitals were included from all regions to obtain a representative sample. Purposeful sampling was done by including 45 patients from each hospital. Patients were included in the study if they had any disease listed in the Charlson comorbidity index (CCI), visiting outpatient for routine follow-up, prescribed with at least two drugs, age above 20 years, and with available medical records. Patients with diseases other than listed in CCI were excluded. Systematic random sampling method was used to avoid biases. Every third patient visiting the outpatient clinic was invited to participate in the study. The study protocol was approved by the Research and Ethics committee of the Faculty of Pharmacy, Bahauddin Zakariya University, and the concerned hospitals (registration no. Bzbp-18-4207). Written informed consent was obtained from all the participants.

### Data Collection

A self-developed questionnaire was used to collect the patients' demographic data, socioeconomic status, clinical condition, diagnosis, names, number and doses of prescribed medicines, self-medication, if any, route of administration, appropriate drug selection, drug–disease interaction, and drug–drug interactions. The data collection form was filled after physician consultation. Medication errors were defined as inappropriate dose (dosage too low or too high), invalid indication (drug prescribed without clinical indication), drug duplication (multiple drugs for the same condition), inappropriate dosing frequency (too short or too long dosing frequency), longer duration of drug therapy than recommended, and drug–drug interactions (combination in the prescription which is prohibited or may cause harm to the patient) (29). A patient utilizing five or more medicines was considered to be a polypharmacy patient (30). The comorbidities were scored using the CCI scale (31). Overburdened was considered to check 20 or more patients in an hour (32). Data collection was done during December 2017 to August 2018 by three graduating class pharmacy students.

### Drug–Drug Interaction Software

For identification of medication errors, Pharmacotherapy: a pathophysiologic approach, 7th edition, Lexicomp Drug Information Handbook, 22nd edition, Harrison's principles of internal medicine, 17th edition, and Applied therapeutics: the clinical use of drugs, Uptodate®2009, were used. The possible potential hazardous combinations of drugs were evaluated using a drug interaction software, Micromedex® (Thomson Reuters Healthcare Inc., Greenwood Village, CO, USA) (33). For fixed drug combinations, each active ingredient was treated separately. All drugs in a prescription were added one by one in the software. The software output displays all the possible interacting combination(s) in a prescription and also provides information about the possible adverse effects of an interaction. Micromedex classifies the interactions as contraindicated (the drug pair is strictly prohibited and may be life-threatening), major (have a risk of any organ damage), moderate (require a change in therapy and/or monitoring), and minor (cause increased side effects and can be avoided by adjusting the dose) drug–drug interactions (34). All the errors were reviewed by the hospital physician and clinical pharmacist.

## Data Analysis

Frequencies and percentages were reported for categorical variables, and mean and standard deviation were reported for continuous variables. Normality of the data was checked using Shapiro–Wilk test of normality. To assess the face validity, a committee of experts including two academician, two medical practitioners, and a clinical pharmacist critically reviewed the questionnaire. Internal consistency of the questionnaire was assessed based on the value of Cronbach's alpha coefficient. Internal consistency is considered as excellent if Cronbach's α is ≥0.9, strong if Cronbach's α is ≥0.8, acceptable if Cronbach's α is ≥0.7, and reasonable if Cronbach's α is ≥0.6 (35). Univariate and multivariate logistic regression analyses were used to assess the impact of age ≥60 years, overburdened healthcare system, polypharmacy, number of comorbidities, CCI score, prescription by a specialist, trainee practitioner, multiple prescribers to one patient, presence of previous medical record, review of prescription by a clinical pharmacist, and use of online software in prescription generation on the prevalence of medication errors in patients suffering from chronic diseases. Initially, a univariate analysis was performed, and variables with univariate *p* < 0.02 were included in the multiple regression model. Odd ratios were used to assess the influence of different risk factors. The Hosmer–Lemeshow test was used to check the goodness-of-fit of the model. A *p*-value of 0.05 or less was considered as statistically significant. All analyses were performed using SPSS version 24.0 (IBM SPSS Statistics for Windows, 142 Armonk, NY: IBM Corporation).

## Results

Out of 803 patients, 41.09% were male and 58.91% were females. The mean age was reported as 48.34 (9.4) years. The average number of medicines per encounter was 5.23 (2.8). More than five drugs were prescribed in almost 73.12% patients. The questionnaire showed good reliability with Cronbach's alpha coefficient value of 0.71. The most frequent diagnosis was diabetes mellitus (38.27%), followed by myocardial infarction (28.78%), peripheral vascular disease (22.54%), peptic ulcer disease (21.23%), liver disease (17.32%), congestive heart failure (13.43%), COPD (11.23%), hemiplegia (9.23%), moderate to severe chronic kidney disease (9.2%), connective tissue disease (6.3%), cerebrovascular disease (6.2%), dementia (3.9%), solid tumor (2.3%), malignant lymphoma (1.2%), and leukemia (0.12%). The demographic and clinical data of patients are shown in Table 1. Different errors found in the prescribing were inappropriate dose (39.75%), invalid indication (43.17%), multiple drugs for the same condition (39.3%), longer duration of drug therapy than recommended (12.6%), and drug–drug interactions (DDIs) (43.58%). Overall, 38.25% of prescriptions showed one or two and 17.23% prescriptions showed three or more than three drug–drug interaction regardless of severity. Among drug–drug interactions, 4.13% were contraindicated, 22.8% were severe, 53.42% were moderate, and 19.8% were minor. The medication errors in the prescription are presented in Table 2.

**Table 1:** Demographic and clinical characteristics of the patients included in the study (*N* = 803).

**Variables**	**Results**
Gender, male	41.09% (330)
Mean age	48.34 ± 9.4
Patients age ≥60 years	36.98% (297)
Mean number of prescribed drugs	5.23 (2.8)
Patients prescribed with five or more drugs	73.12%
Mean number of physicians who prescribed to single patient	1.67 ± 0.73
Mean number of diseases per patient	1.8 ± 0.81
Patients with more than two diseases	44.20% (355)
CCI	1.73 ± 2.44
**Diagnosis**	
Diabetes mellitus	38.27%
Moderate to severe CKD	9.2%
Connective tissue disease	6.3%
COPD	11.23%
Liver disease	17.32%
Peripheral vascular disease	22.54%
Myocardial infarction	28.78%
Congestive heart failure	13.43%
Cerebrovascular disease	6.2%
Hemiplegia	9.23%
Peptic ulcer disease	21.23%
Dementia	3.9%
Leukemia	0.12%
Malignant lymphoma	1.2%
Solid tumor	2.3%
AIDS	–

*Data are presented as mean ± standard deviation and percentage (number) unless otherwise stated*.

*COPD, chronic obstructive pulmonary disease; CCI, Charlson comorbidity index; CKD, chronic kidney disease; AIDS, acquired immunodeficiency syndrome*.

**Table 2:** Prevalence of medication errors in chronic disease patients.

**Medication errors**	**Percentage**
Inappropriate dose	39.75%
Invalid indication	43.17%
Multiple drugs for same condition	39.3%
Too short dosing frequency	28.1%
Longer duration of drug therapy than recommended	12.6%
**Drug–drug interactions (DDI)**	
Overall DDI	43.58%
Minor DDI	19.8%
Moderate DDI	53.42%
Severe DDI	22.8%
Contraindicated DDI	4.13%
**Mean drug–drug interactions per patient**	
Minor DDI	0.41 ± 0.3
Moderate DDI	1.87 ± 2.03
Severe DDI	1.36 ± 1.96
Contraindicated DDI	0.14 ±.39
Presence of one to two DDI	38.25%
Presence of three or more DDI	17.23%

*Overall prevalence, presence of at least one DDI regardless of type and severity*.

*Percentages may not add up to overall prevalence as many patients were having more than one DDI*.

### Risk Factors of Medication Errors in Patients Suffering From Chronic Diseases

Independent variables showing a significant relationship with medication errors in univariate analysis (*p* < 0.1) were added in the multiple logistics regression. The odds ratio (OR), 95% confidence intervals, and *p*-value for the independent variables included in the multiple logistic regression are presented in Table 3. A greater risk for the occurrence of medication errors was associated with age ≥60 years (OR = 1.9; 95% CI = 1.3–3.1; *p* = 0.001), overburdened healthcare system (≥20 patients in 1 h; OR = 2.2; 95% CI = 1.64–3.56; *p* < 0.000), number of prescribed drugs ≥5 (OR = 1.74; 95% CI = 1.02–2.64; *p* < 0.000), number of comorbidities (OR = 2.6; 95% CI = 1.72–3.6; *p* = 0.003), CCI score (OR = 1.31; 95% CI = 0.49–1.84; *p* = 0.004), multiple prescribers to one patient (OR = 1.12; 95% CI = 0.64–1.76; *p* = 0.001), and trainee practitioner (OR = 1.03; 95% CI = 0.61–1.65; *p* = 0.003).

**Table 3:** Multiple logistic regression analysis for risk factors associated with medication errors in chronic disease patients.

**Variables**	**OR**	**95% CI**	***p*-value**
Gender, male	0.72	0.52–1.21	0.7
Age ≥60 years	1.9	1.3–3.1	0.001
Number of prescribed drugs ≥5	1.74	1.02–2.64	0.000
Overburden	2.2	1.64–3.56	0.000
Comorbidities	2.6	1.72–3.6	0.003
CCI score	1.31	0.49–1.84	0.004
Multiple prescribers to one patient	1.12	0.64–1.76	0.001
Trainee practitioner	1.03	0.61–1.65	0.003
Prescription by a specialist	0.27	0.03–0.47	0.4
Presence of previous medical record	0.63	0.17–1.34	0.09
Use of online software in prescription generation	0.69	0.23–1.39	0.1
Review of prescription by a clinical pharmacist	0.28	0.03–0.59	0.7
Private hospitals	1.24	1.01–1.52	0.039

*OR, odds ratio; CCI, Charlson comorbidity index; Overburden, ≥20 patients in 1 h*.

*A p-value of 0.05 or less was considered as statistically significant*.

The association was not significant with the patients' gender (OR = 0.72; 95% CI = 0.52–1.21; *p* = 0.7), prescription by a specialist (OR = 0.27; 95% CI = 0.03–0.47; *p* = 0.4), presence of previous medical record (OR = 0.63; 95% CI = 0.17–1.34; *p* = 0.09), use of online software in prescription generation (OR = 0.69; 95% CI = 0.23–1.39; *p* = 0.1), and review of prescription by a clinical pharmacist (OR = 0.28; 95% CI = 0.03–0.59; *p* = 0.7).

The logistic regression model showed an accuracy of 64.73%. The logistic regression model showed good model fit, as demonstrated by the non-significant difference (*p* = 0.914) between observed and predicted probabilities in the Hosmer–Lemeshow test and a significant difference (*p* < 0.001) between observed and predicted probabilities in the goodness of fit model test.

## Discussion

Medication errors result in increasing burden on the healthcare system. The factors associated with medication errors were studied in different hospitals of South Punjab, Pakistan. Consistent with previous literature, older age, overburdened healthcare system, higher number of prescribed drugs, and comorbidities were significantly associated with an increase in the risk of medication errors (21, 36, 37). The number of medicines in the prescription was the most significant risk factor for inappropriate prescribing. Polypharmacy was considered as the utilization of five or more drugs. WHO recommends a maximum of three drugs in a prescription as optimal therapy (38). More than five drugs were prescribed to a lot of number of patients. It is a well-recognized risk factor for medication errors (8, 36, 39). The risk of error increased from 17 to 66% with the increase in the number of drugs in a prescription from two to 16 (40). Polypharmacy requires special attention. Potentially inappropriate drugs should be identified, and drugs with narrow therapeutic ranges or associated with frequent adverse drug reactions should be avoided if possible and carefully monitored when used.

Comorbidities were associated with an increased risk of medication errors. Comorbidities increase the interaction of patients with healthcare professionals, and patients use more medicines for a longer duration (21). Comorbidities were associated with the increase in the risk of medication errors. In a country like Pakistan, a complete electronic medical record of a patient is not available. Patients visit to different healthcare professionals for different comorbidities. This causes confusion and lack of medical records from one prescriber, resulting in medication error from other prescribers due to a lack of knowledge of the medication history (21). Workload was also significantly associated with an increase in the risk of medication errors. Overburden and fatigue can affect the efficiency of health professionals. Reducing the workload may have positive effects in avoiding medication errors. A study showed that reducing the workload on a physician was positively associated to avoiding the risk of medication errors (41).

It was observed that prescription from a specialist and review of a prescription by a consultant can reduce the chances of medication errors. This may be due to the fact that healthcare professionals are usually more familiar with the errors related to the drugs they more frequently prescribe. Specialists mostly prescribe drugs of specific categories, so they are more aware of them, and the chances of error decrease. Moreover, senior physicians learn a lot from their experience, so the chances of error reduced with the experience. Our findings are in line with the previous literature. Hussain et al., in their study, found that prescription from practitioners who had seen the patients with potential DDIs before contained less errors and DDIs (42). Results of another study showed that prescriptions from a specialist contained less DDIs than prescriptions from general practitioners (43). Regular training of the junior medical staff can reduce the rate of potential medical errors. Training and feedback control of prescribing by senior doctors should be associated with the availability of online references for immediate identification and verification of potential prescribing faults (44). Moreover, the choice of treatment should generally be in line with approved guidelines, although flexibility may be necessary in individual cases (45). Prescription must be reviewed by a senior healthcare professional to remove medical errors that may be mistaken by the prescriber.

The review of prescription by a clinical pharmacist also reduced the risk of errors. The importance of a clinical pharmacist on the healthcare system is well-recognized, but unfortunately in Pakistan, the role of a clinical pharmacist is not well-established. Most of the basic healthcare units and private hospitals do not have a pharmacist. In teaching hospitals, pharmacists are working on administrative posts instead of in clinical practice. Literature shows that the involvement of a clinical pharmacist in a health system reduces the risk of errors, increases adherence to therapy, and supports physicians to manage drug-related events (46, 47). Another study demonstrated that most of the drug-related events identified by clinical pharmacists were not detected in routine clinical practice (48).

The association was also not significant with the use of online software programs in prescription generation. The manual procedure for prescription generation is associated with a greater risk of medication errors. Healthcare professionals have to know about a large number of potential DDIs and their adverse effects. Continuous progression in medical education requires continuous learning. The use of information technology (IT) can overcome this issue. IT can be used to learn about potential drug-related events, to check a prescription for potential DDIs, and to get access to a patient's medical records (49). A previous study demonstrated that healthcare professionals who rely on personal digital assistance and computerized alert system to check medication-related errors were less prone to inappropriate prescribing (50). A study showed that the use of computer software programs during the prescribing process reduced the chances of error up to 66% in general physicians (51). Ansari and Neupane compared the manual and the computer-generated prescriptions in hospitals of Nepal. Hospitals wherein the online drug interaction system was installed were less prone to medication errors (52).

Due to the increasing cost of healthcare services, there is an urgent need to consider medication errors in routine clinical practice to reduce the burden on the healthcare system. Reduction of complexity in the act of prescribing by the introduction of automation improves a prescriber's knowledge by education, use of online aid, feedback control systems, and monitoring of the effects of interventions that can help in the reduction of medication errors. Most of the medication errors are preventable; medication review of the prescriptions by an experienced healthcare professional can detect these errors, and reconciliation can help remove such errors before these reach the patient.

This study is the first to investigate the risk factors for medication errors in chronic disease patients visiting as outpatients for regular follow-up. Currently, there is no system in South Punjab to report medication errors. Therefore, policy makers and healthcare providers are encouraged to develop and implement medication error reporting forms. Despite several strengths, few limitations should be kept in mind before interpretation of the results. This study was conducted in a specific population (patients suffering from chronic diseases), so its results should be generalized with caution, but it can be assumed that the results found in this study can be generalized to chronic disease patients. Moreover, the frequency of potential medication errors was assessed, but their impact on other outcomes like outpatient visits, hospital admissions, and quality of life was not assessed. In a detailed study, the impact of medication errors on patient-relevant endpoints such as health status, quality of life, and hospital admissions should be assessed.

## Conclusion

A greater risk for the occurrence of medication errors was associated with older age, overburdened healthcare system (≥20 patients in 1 h), higher number of drugs in a prescription, comorbidities, multiple prescribers to one patient, and trainee practitioner. The risk was not significantly associated with a patient's gender, prescription by a specialist, presence of previous medical record, use of online software in prescription generation, and review of prescription by a clinical pharmacist.

## Implications for Policy and Practice

Due to the increasing cost of healthcare services, there is an urgent need to consider medication errors in routine clinical practice to reduce the burden on the healthcare system. Reduction of complexity in the act of prescribing by the introduction of automation, improving the prescriber's knowledge by education, use of online aid and feedback control systems, and monitoring the effects of interventions can help in the reduction of medication errors.

Knowledge about the risk factors of potential drug–drug interactions and designing and implementing strategies to control them can reduce the economic and humanistic burden due to drug-related events.

## Data Availability Statement

The datasets generated for this study are available on request to the corresponding author.

## Ethics Statement

The study protocol was approved by the Research and Ethics committee of Faculty of Pharmacy Bahauddin Zakariya University and the concerned hospitals (registration no. Bzbp-18-4207). The patients/participants provided their written informed consent to participate in this study.

## Author Contributions

MR, AR, II, SA, IK, SShah, GA, SShak, MA, and KH made substantial contributions to the conception and design of the study and the analysis and interpretation of the data. MR and AR made substantial contributions to the analysis and interpretation of the data. All the authors drafted the work or revised it critically for important intellectual content, reviewed, critiqued, and approved the final version submitted for publication.

## Conflict of Interest

The authors declare that the research was conducted in the absence of any commercial or financial relationships that could be construed as a potential conflict of interest.
